# Supplementary material for: A PDCD1 Role in the Genetic Predisposition to NAFLD-HCC?
Source: Cancers (Basel). 2021 Mar 19;13(6):1412. doi: 10.3390/cancers13061412 (PMC8003582; doi:10.3390/cancers13061412)
Supplement: Supplementary file 1 [file cancers-13-01412-s001.pdf]

cancers

MDPI

Article

A *PDCD1* Role in the Genetic Predisposition to NAFLD-HCC?

Nardeen Eldafashi, Rebecca Darlay, Ruchi Shukla, Misti Vanette McCain, Robyn Watson, Yang Lin Liu, Nikki McStraw, Moustafa Fathy, Michael Atef Fawzy, Marco YW Zaki, Ann K Daly, João P Maurício, Alastair D Burt, Beate Haugk, Heather J Cordell, Cristiana Bianco, Jean-François Dufour, Luca Valenti, Quentin M Anstee and Helen L Reeves

Citation: Eldafashi, N.; Darlay, R.; Shukla, R.; McCain, M.V.; Watson, R.; Liu, Y.L.; McStraw, N.; Fathy, M.; Fawzy, M.A.; Zaki, M.Y.W.; et al. A *PDCD1* Role in the Genetic Predisposition to NAFLD-HCC? *Cancers* **2021**, *13*, 1412. https://doi.org/10.3390/cancers13061412

Academic Editor: Roland Kappler

Received: 11 February 2021
Accepted: 16 March 2021
Published: date

Publisher's Note: MDPI stays neutral with regard to jurisdictional claims in published maps and institutional affiliations.

Copyright: © 2021 by the authors. Submitted for possible open access publication under the terms and conditions of the Creative Commons Attribution (CC BY) license (http://creativecommons.org/licenses/by/4.0/).

Supplementary Materials:

Table S1. Applied Biosciences Taqman Assays.

SNP ID	Assay ID
MICA rs2596542	C_27301153_10
CD44 rs187115	C_779820_10
PDCD1 rs7421861	C_26891639_10
PDCD1 rs10204525	C_172862_10
PNPLA3 rs738409	C_7241_10
TM6SF2 rs2596542	C_89463510_10

Table S2. Calculations to estimate the power of our cohorts to evaluate the genetic variation on HCC risk in NAFLD.

SNP ID	% Prevalence of HCC in NAFLD	MAF	Allelic RR	Power (for detecting effect at $p = 0.05$) with 391 cases and 594 controls	Power (for detecting effect at $p = 0.05$) with 198 cases and 416 controls
Calculations of power to detect an allelic relative risk greater than ≥1.3					
PNPLA3 rs738409	0.5	0.419	1.3	0.8131	0.5774
TM6SF2 rs2596542	0.5	0.119	1.45	0.8120	0.5843
MICA rs2596542	0.5	0.332	1.3	0.7876	0.5517
CD44 rs187115	0.5	0.344	1.3	0.7930	0.5571
PDCD1 rs7421861	0.5	0.332	1.3	0.7876	0.5517
PDCD1 *rs10204525*	0.5	0.095	1.5	0.8131	0.5870
Calculations of power to detect an allelic relative risk greater than ≥1.4					
PNPLA3 rs738409	0.5	0.419	1.4	0.9552	0.7900
TM6SF2 rs2596542	0.5	0.119	1.6	0.9563	0.8023
MICA rs2596542	0.5	0.332	1.4	0.9446	0.7689
CD44 rs187115	0.5	0.344	1.4	0.9470	0.7737
PDCD1 rs7421861	0.5	0.332	1.4	0.9446	0.7689
PDCD1 rs10204525	0.5	0.095	1.65	0.9485	0.7867
Calculations of power if HCC prevalence was in keeping with that for NAFLD-cirrhosis					
Calculations of power to detect an allelic relative risk greater than ≥1.3					
PNPLA3 rs738409	2.6	0.419	1.3	0.8293	0.5957
TM6SF2 rs2596542	2.6	0.119	1.45	0.8295	0.6043
MICA rs2596542	2.6	0.332	1.3	0.8049	0.5700
CD44 rs187115	2.6	0.344	1.3	0.8100	0.5754
PDCD1 rs7421861	2.6	0.332	1.3	0.8049	0.5700
PDCD1 *rs10204525*	2.6	0.095	1.5	0.8307	0.6073
Calculations of power to detect an allelic relative risk greater than ≥1.4					
PNPLA3 rs738409	2.6	0.419	1.4	0.9622	0.8070
TM6SF2 rs2596542	2.6	0.119	1.6	0.9638	0.8207
MICA rs2596542	2.6	0.332	1.4	0.9529	0.7868
CD44 rs187115	2.6	0.344	1.4	0.9550	0.7914
PDCD1 rs7421861	2.6	0.332	1.4	0.9529	0.7868

PDCD1 *rs10204525*	2.6	0.095	1.65	0.9571	0.8060

Legend: These power calculations were performed comparing 392 cases with 594 controls, or 199 cases with 416 controls, using GPC software (http://zzz.bwh.harvard.edu/gpc/) and the case control for discrete traits option. The minor allele frequencies (MAF) for the SNPs in control cases with NAFLD are as described in Supplementary Table 6. We have estimated the prevalence of HCC with a control population like ours to be 0.5%, although in cirrhotic cases this may be as high as 2.6% [1]. Calculations for the two estimates of prevalence, alongside relative risk (RR) of ≥1.3 or ≥1.4 are shown.

Table S3. Changes in association with TNM Stage.

Table S3A: The decline in BMI through different TNM stages in NAFLD-HCC patients compared to the control group (Newcastle cohort).

	**TNM stage**	**NAFLD (n = 407)**	**NAFLD-HCC (n = 190)**	***p*-Value**	
**BMI**	0	35.031±0.28		<0.0001[1]	<0.002[2]
	I		32.38± 0.67		
	II		34.11±0.83		
	III		29.99±0.78		
	IV		29.01±1.63		

BMI—body mass index; TNM—Tumour Node Metastases; p-value estimated by Kruskal-Wallis test. In a small number of cases BMI was not available. [1]significance across all stages including controls. [2]significance in cancer cases only.

Table S3B: comparison of BMI values between NAFLD and NAFLD-HCC patients according to the cirrhotic changes in their livers.

	**Non-cirrhotic NAFLD**			**Cirrhotic NAFLD**		
**TNM stage**	**Control**	**HCC**	**Number**	**Control**	**HCC**	**Number**
0	34.20		353	36.85		63
I		30.90***	37		32.05*	43
II		33.50	5		34.00	44
III		26.30	28		33.00	27
IV		25.00	7		32.00	8

p-values for TNM Stage I compared to controls. ***<0.0001; **p* = 0.014. Mann-Whitney Test.

Table S4. Demographic characteristics of Newcastle cirrhotic and NC NAFLD-HCC cohort.

**Newcastle NAFLD-HCC**		**All n = 198 (%)**	**Cirrhosis n = 121 (%)**	**No-cirrhosis n = 77 (%)**	***p*-Value**
**Age (years, median)**		73	71	76	<0.0001
Gender	Male	157 (79.3)	92 (76.0)	65 (84.4)	0.156
	female	41 (20.7)	29 (24.0)	12 (15.6)	
BMI (median)	All	31.3	33.0	28.6	<0.0001
	TNM I	31.4	32.1	30.9	
	TNM II	34.0	34.0	33.5	
	TNM III	30.0	33.0	26.3	
	TNM IV	28.0	32.0	25.0	
T2DM	absent	60 (30.3)	29 (24.0)	31 (40.3)	0.015*
	present	138 (69.7)	92 (76.0)	46 (59.7)	
Albumin (median)		41.0	39.0	43.0	<0.0001
Bilirubin (median)		12.0	13.0	9.0	<0.0001
PT (median)		13.0	13.0	12.0	<0.0001
Ascites	absent	170 (85.9)	98 (81.0)	72 (93.5)	0.014*
	present	28 (14.1)	23 (19.0)	5 (6.5)	
Encephalopathy	absent	189 (95.5%)	112 (92.6)	77 (100.0)	0.014*

	present	9 (4.5)	9 (7.4)	0 (0.0)	
Tumour number (med)		2.16 (1)	2.29 (2)	1.95 (1)	0.111
Tumour size cm (med)		5.65 (4.0)	4.48 (3.5)	7.44 (6.0)	<0.0001
PVT	absent	163 (82.3)	100 (82.6)	63 (81.8)	1.00
	present	35 (17.7)	21 (17.4)	14 (18.2)	
EHD	absent	179 (90.4)	111 (91.7)	68 (88.3)	0.585
	present	19 (9.6)	10 (8.2)	9 (11.7)	
AFP (median)		3755 (7)	3385 (7)	4340 (6)	0.532
TNM Stage	I	80 (40.4)	43 (35.5)	37 (48.1)	<0.0001
	II	49 (24.7)	44 (36.4)	5 (6.5)	
	III	55 (27.8)	27 (22.3)	28 (36.4)	
	IV	14 (7.1)	7 (5.8)	7 (9.1)	
BCLC Stage	0+A	49 (24.7)	34 (27.1)	15 (19.5)	0.522
	B	30 (15.2)	18 (14.9)	12 (15.6)	
	C	101 (51.0)	57 (47.1)	44 (57.1)	
	D	18 (9.0)	12 (9.9)	6 (7.8)	
Histology differentiation	Well	31 (33.3)	11 (33.3)	20 (33.3)	0.470
	moderate-poor	48 (51.6)	15 (45.5)	33 (55.0)	
	poor	14 (15.1)	7 (21.2)	7 (11.7)	
Histology Architecture	Trabecular	48 (55.2)	14 (45.2)	34 (55.7)	0.562
	Solid/compact	32 (34.8)	11 (35.5)	21 (34.4)	
	Pseudoglandular	5 (5.4)	2 (6.5)	3 (4.9)	
	Mixed/cholangio	7 (7.6)	4 (12.9)	3 (4.9)	
Histology Subtype	No subtype	57 (63.3)	18 (58.1)	39 (66.1)	0.552
	SH-HCC	23 (25.6)	11 (35.5)	12 (20.3)	
	Clear Cell	3 (3.3)	1 (3.2)	2 (3.4)	
	Macrotrabecular Massive	0	0	0	
	Scirrhous	3 (3.3)	1 (3.2)	2 (3.4)	
	Chromophobe	0	0	0	
	Fibrolamellar	3 (3.3)	0	3 (5.1)	
	Neutrophil rich	0	0	0	
	Lymphocyte rich	1 (1.1)	0	1 (1.7)	
Treatment	OLTx	6 (3.0)	6 (5.0)	0 (0.0)	<0.0001
	Resection	12 (6.1)	2 (1.7)	10 (13.0)	
	Ablation	22 (11.1)	18 (14.9)	4 (5.2)	
	Arterial	85 (42.9)	56 (46.3)	29 (37.7)	
	Medical	17 (8.6)	11 (9.1)	6 (7.8)	
	Supportive	56 (28.3)	28 (23.1)	28 (36.4)	

Table S5. Features associated with survival in NAFLD-HCC patients **(Newcastle)**.

Variable		Univariate Analysis		Multivariate Analysis		
		p-Value	HR	*p*-Value	HR	Confidence
Age		<0.0001	1.034	0.006	1.071	1.020-1.124
Gender		0.717				
BMI		0.010		0.225		
T2DM		0.050	0.709	0.110		
Cirrhosis		0.791				
Tumour Number		<0.0001	1.159	<0.0001	1.356	1.158-1.588
Tumour Size		<0.0001	1.068	0.002	1.122	1.042-1.208
Extra hepatic disease		<0.0001	0.316	0.689		
Portal Vein Thrombus		<0.0001	0.317	0.002	0.207	0.078-0.550
Ascites		<0.0001	0.244	0.163		
Encephalopathy		0.199				
ECOG PST	0	<0.0001		0.030		
	1	0.001	0.250	0.051	0.115	0.013-1.011

	2	0.022	0.395	0.063	0.121	0.013-1.118
	3 (reference)	0.266	0.626	0.319	0.320	0.034-3.007
Prothrombin Time		0.085				
Albumin		<0.0001	0.914	0.097		
Bilirubin		<0.0001	1.109	0.763		
AFP		<0.0001	1.000	0.238		
Histology differentiation	well	0.001		0.004		
	moderate	<0.0001	0.257	0.001	0.161	0.054-0.481
	poor (reference)	0.11	0.597	0.047	0.412	0.172-0.988

Table S6. Genotype frequencies of the candidate SNPs in Newcastle NAFLD and NAFLD-HCC.

		**NAFLD Controls**		**NAFLD-HCC**	
		**Number (%)**		**Number (%)**	
PNPLA3 rs738409	CC	170	(40.9%)	67	(33.8%)
	CG	184	(44.2%)	85	(42.9%)
	GG	62	(14.9%)	46	(23.2%)
TM6SF2 rs58542926	CC	323	(77.6%)	132	(66.7%)
	CT	85	(20.4%)	60	(30.3%)
	TT	8	(1.9%)	6	(3.0%)
MICA rs2596542	CC	183	(44.0%)	92	(46.9%)
	TC	190	(45.7%)	89	(45.4%)
	TT	43	(10.3%)	15	(7.7%)
CD44 rs187115	TT	175	(42.1%)	79	(39.9%)
	CT	197	(47.4%)	96	(48.5%)
	CC	44	(10.6%)	23	(11.6%)
PDCD1 rs7421861	AA	180	(43.3%)	126	(63.6%)
	GA	189	(45.4%)	53	(26.8%)
	GG	47	(11.3%)	19	(9.6%)
PDCD1 rs10204525	CC	345	(82.9%)	154	(78.6%)
	TC	66	(15.9%)	38	(19.4%)
	TT	5	(1.2%)	4	(2.0%)

Table S7. Dominant, recessive and genotype associations with HCC risk assessed by logistic regression in the Newcastle cohort.

**Dominant Model**		**Identity**	***p* Value**	**OR**
PNPLA3	C>G	rs738409	0.104	1.341
TM6SF2	C>T	rs58542926	3.90E-03	1.737
MICA	T>C	rs2596542	0.535	0.898
CD44	C>T	rs187115	0.484	1.132
PDCD1	A>G	rs7421861	2.195E-06	0.430
PDCD1	C>T	rs10204525	0.185	1.334
		Recessive Model		
PNPLA3	C>G	rs738409	0.01094	1.739
TM6SF2	C>T	rs58542926	0.3943	1.594
MICA	T>C	rs2596542	0.3003	0.7229
CD44	C>T	rs187115	0.6841	1.118
PDCD1	A>G	rs7421861	0.5378	0.838
PDCD1	C>T	rs10204525	0.422	1.721
		Genotype Model		
PNPLA3	C>G	rs738409	0.02997	
TM6SF2	C>T	rs58542926	0.01546	
MICA	T>C	rs2596542	0.5537	
CD44	C>T	rs187115	0.7653	
PDCD1	A>G	rs7421861	7.837e-6	
PDCD1	C>T	rs10204525	0.3717	

Table S8. Minor Allele Frequency (MAF) for the candidate genes in the Newcastle NAFLD GWAS study, reporting any difference (p value) compared to NAFLD GWAS controls (without NAFLD) .

	SNP	Chromosome	maf	*p*-Value	OR	se
PDCD1	rs7421861	2	0.3548	0.503259	0.9683	0.048125
MICA	rs2596542	6	0.3235	0.102425	1.054	0.032202
CD44	rs187115	11	0.3484	0.798905	1.005	0.019577
TM6SF2	rs58542926	19	0.06636	2.05E-11	1.609	0.070964
PNPLA3	rs738409	22	0.2183	1.45E-49	1.827	0.040716

Table S9. Prevalence of PDCD1rs10204525 SNP in Newcastle NAFLD versus NAFLD-HCC patients —gender comparison.

		**Male**		**Female**	
		**Control**	**HCC**	**Control**	**HCC**
		**Number (%)**	**Number (%)**	**Number (%)**	**Number (%)**
***PDCD1* rs10204525**	CC	191 (82.3)	128 (82.6)	154 (83.7)	26 (63.4)
	TC	39 (16.8)	24 (15.5)	27 (14.7)	14 (34.1)
	TT	2 (0.9)	3 (1.9)	3 (1.6)	1 (2.4)

Table S10. Linkage disequilibrium analysis for PDCD1 rs7421861 A>G and rs10204525 C>T.

Haplotype	Frequency	Expectation under LE
TG	0	0.030488
CG	0.304878	0.274390
TA	0.1	0.069512
CA	0.595122	0.625610

In phase alleles are TA/CG. R-sq = 0.0487329 D' = 1.

Table S11. Allelic analyses restricted to 353 NAFLD controls and 78 NAFLD-HCC cases without cirrhosis.

**Gene**		**Identity**	***p* Value**	**OR**	**Conditioned on PNPLA3 + TM6SF2**	
					***p* Value**	**OR**
PNPLA3	C>G	rs738409	0.9086	1.02		
TM6SF2	C>T	rs58542926	0.2607	1.34		
MICA	T>C	rs2596542	0.8134	1.05	0.8239	1.04
CD44	C>T	rs187115	0.2943	1.22	0.386	1.18
PDCD1	A>G	rs7421861	5.214e-3	0.56	6.841e-3	0.57
PDCD1	C>T	rs10204525	0.1111	1.51	0.159	1.442
			Age, Sex, T2DM			
PNPLA3	C>G	rs738409	0.6912	1.13		
TM6SF2	C>T	rs58542926	0.1895	0.54		
MICA	T>C	rs2596542	0.8175	1.08	0.6734	1.15
CD44	C>T	rs187115	0.4658	1.27	0.4891	1.26
PDCD1	A>G	rs7421861	0.02129	0.46	0.02487	0.47
PDCD1	C>T	rs10204525	0.1524	1.83	0.1334	1.92

Table S12. Genotype frequencies of the candidate SNPs in Berne and Milan NAFLD and NAFLD-HCC **cohorts.**

		**Berne**				**Milan**			
		**NAFLD controls**		**NAFLD-HCC**		**NAFLD controls**		**NAFLD-HCC**	
		**Number (%)**		**Number (%)**		**Number (%)**		**Number (%)**	
PNPLA3 rs738409	CC	22	(28.9%)	25	(29.8%)	26	(25.5%)	23	(21.1%)
	CG	30	(39.5%)	33	(39.3%)	42	(41.2%)	48	(44.0%)
	GG	24	(31.6%)	26	(31.0%)	34	(33.3%)	38	(34.9%)
TM6SF2	CC	58	(76.3%)	61	(72.6%)	86	(84.3%)	91	(83.5%)

rs58542926	CT	12	(15.8%)	20	(23.8%)	16	(15.7%)	16	(14.7%)
	TT	6	(7.9%)	3	(3.6%)	0	(0.0%)	2	(1.8%)
PDCD1 rs7421861	AA	62	(81.6%)	65	(78.3%)	82	(80.4%)	94	(86.2%)
	GA	14	(18.4%)	17	(20.5%)	17	(16.7%)	15	(13.8%)
	GG	0	(0.0%)	1	(1.2%)	3	(2.9%)	0	(0.0%)
PDCD1 rs10204525	CC	36	(47.4%)	40	(47.6%)	47	(46.1%)	45	(41.3%)
	TC	34	(44.7%)	34	(40.5%)	45	(44.1%)	49	45.0%)
	TT	6	(7.9%)	10	(11.9%)	10	(9.8%)	15	13.8%)

Table S13. Statistical analysis of the candidate SNPs in Berne and Milan NAFLD and NAFLD-HCC **cohorts.**

**Gene**		**Identity**	**Berne**		**Milan**	
			**pvalue**	**OR**	**p value**	**OR**
PNPLA3	C>G	rs738409	0.9069	0.98	0.5666	1.11
TM6SF2	C>T	rs58542926	0.9449	0.98	0.6299	1.18
PDCD1	A>G	rs7421861	0.7187	1.09	0.3464	1.21
PDCD1	C>T	rs10204525	0.5043	1.28	0.1335	0.60

Table S14. Table shows the eQTL effect size and significance for PDCD1 rs7421861 and indicated gene expression in specific tissues or cell types.

**rs7421861**				
**Gene**	**Symbol**	***p*-Value (-log10)**	**Effect size**	**Tissue/Cell type**
ENSG00000154252	GAL3ST2	2.106816573	0.893287	macrophage_Salmonella
ENSG00000188011	RTP5	3.34931761	−0.593432	monocyte_R848
ENSG00000188389	PDCD1	4.672716691	−0.521107	monocyte_R848
ENSG00000204099	NEU4	3.122583154	0.58719	monocyte
ENSG00000204099	NEU4	6.98505965	−0.840789	macrophage_Listeria
ENSG00000215692	AC114730.8	1.574226201	0.551741	macrophage_Salmonella
ENSG00000224272	AC114730.3	2.768008245	−0.525108	monocyte_R848
ENSG00000224272	AC114730.3	2.441329822	−0.540187	macrophage_Listeria
ENSG00000235151	AC131097.3	1.996448999	0.567401	monocyte
ENSG00000235151	AC131097.3	1.513586525	0.520945	macrophage_Salmonella

Table S15. Table shows the eQTL effect size and significance for PDCD1 rs10204525 and indicated gene expression in specific tissues or cell types.

**rs10204525**				
**Gene**	**Symbol**	***p*-Value (-log10)**	**Effect Size**	**Tissue/Cell Type**
ENSG00000235351	AC114730.4	1.773549611	-0.558197	Tfh_memory
ENSG00000224272	AC114730.3	4.994952543	2.04677	CD4_T-cell_anti-CD3-CD28
ENSG00000224272	AC114730.3	2.762056834	1.19196	CD8_T-cell_anti-CD3-CD28
ENSG00000224272	AC114730.3	6.825815129	1.11348	monocyte_Pam3CSK4
ENSG00000224272	AC114730.3	2.94709454	1.07584	Th1_memory
ENSG00000224272	AC114730.3	5.839852731	1.07347	monocyte_LPS
ENSG00000224272	AC114730.3	2.781146899	0.998949	CD8_T-cell_naive
ENSG00000224272	AC114730.3	6.697188712	0.977589	monocyte_IAV
ENSG00000224272	AC114730.3	2.732894008	0.966898	Th1-17_memory
ENSG00000224272	AC114730.3	6.180712769	0.960048	monocyte_naive
ENSG00000224272	AC114730.3	5.872186001	0.885419	monocyte_R848
ENSG00000224272	AC114730.3	2.037487186	0.861265	Tfh_memory
ENSG00000224272	AC114730.3	1.737084013	0.763997	Th2_memory
ENSG00000180902	D2HGDH	7.181600555	0.809552	monocyte_LPS
ENSG00000180902	D2HGDH	7.951499932	0.802942	monocyte_Pam3CSK4
ENSG00000180902	D2HGDH	7.659242672	0.681617	monocyte_R848
ENSG00000180902	D2HGDH	7.443067024	0.660345	monocyte_naive
ENSG00000180902	D2HGDH	7.577838757	0.63055	monocyte_IAV

ENSG00000234793	AC114730.2	1.655509481	0.590658	CD4_T-cell_anti-CD3-CD28
ENSG00000234793	AC114730.2	3.890819823	0.514726	monocyte_R848
ENSG00000154252	GAL3ST2	6.26525936	1.22017	monocyte_Pam3CSK4
ENSG00000154252	GAL3ST2	4.815183692	1.14643	monocyte_LPS
ENSG00000154252	GAL3ST2	3.441375417	1.13322	CD8_T-cell_anti-CD3-CD28
ENSG00000154252	GAL3ST2	5.18360225	0.978941	monocyte_R848
ENSG00000154252	GAL3ST2	4.402815081	0.898332	monocyte_IAV
ENSG00000154252	GAL3ST2	5.088239593	0.884157	monocyte_naive
ENSG00000154252	GAL3ST2	1.53802752	0.54554	Tfh_memory
ENSG00000215023	AC131097.1	6.281411959	1.09421	monocyte_R848
ENSG00000215023	AC131097.1	6.676743774	0.963099	monocyte_Pam3CSK4
ENSG00000215023	AC131097.1	5.262106371	0.899721	monocyte_LPS
ENSG00000215023	AC131097.1	4.248800216	0.767043	monocyte_IAV
ENSG00000215023	AC131097.1	4.802168307	0.503798	monocyte_naive
ENSG00000204099	NEU4	4.327127571	1.71814	CD4_T-cell_anti-CD3-CD28
ENSG00000204099	NEU4	3.486851679	1.42685	CD8_T-cell_anti-CD3-CD28
ENSG00000204099	NEU4	4.891194895	1.2448	Th2_memory
ENSG00000204099	NEU4	3.951900173	1.22149	Tfh_memory
ENSG00000204099	NEU4	2.57289707	1.10052	monocyte_CD16_naive
ENSG00000204099	NEU4	4.452547752	1.0358	Th17_memory
ENSG00000204099	NEU4	5.76352824	0.942486	monocyte_Pam3CSK4
ENSG00000204099	NEU4	5.6025345	0.88854	monocyte_naive
ENSG00000204099	NEU4	4.47954398	0.846016	monocyte_LPS
ENSG00000204099	NEU4	5.110591838	0.772023	monocyte_IAV
ENSG00000204099	NEU4	2.110257502	0.756715	Th1_memory
ENSG00000204099	NEU4	4.958398114	0.684009	monocyte_R848
ENSG00000204099	NEU4	2.096251471	0.585624	Th1-17_memory
ENSG00000235151	AC131097.3	2.774155935	0.975154	CD4_T-cell_anti-CD3-CD28
ENSG00000235151	AC131097.3	5.655419114	0.837051	monocyte_naive
ENSG00000235151	AC131097.3	4.160501213	0.717407	monocyte_LPS
ENSG00000235151	AC131097.3	2.772578677	0.709116	monocyte_CD16_naive
ENSG00000235151	AC131097.3	4.503774923	0.706194	monocyte_Pam3CSK4
ENSG00000235151	AC131097.3	2.185641181	0.558619	Th17_memory
ENSG00000188389	PDCD1	8.853481275	0.964331	monocyte_Pam3CSK4
ENSG00000188389	PDCD1	8.408514665	0.96187	monocyte_LPS
ENSG00000188389	PDCD1	9.498040203	0.944919	monocyte_naive
ENSG00000188389	PDCD1	2.345629946	0.916861	monocyte_CD16_naive
ENSG00000188389	PDCD1	5.26611451	0.738328	CD4_T-cell_anti-CD3-CD28
ENSG00000188389	PDCD1	7.349027457	0.735695	monocyte_R848
ENSG00000188389	PDCD1	4.761554931	0.591227	CD8_T-cell_anti-CD3-CD28
ENSG00000188389	PDCD1	6.33987668	0.581108	monocyte_IAV
ENSG00000188011	RTP5	11.53507548	1.25191	monocyte_R848
ENSG00000188011	RTP5	11.47059916	1.15669	monocyte_Pam3CSK4
ENSG00000188011	RTP5	9.398728969	1.10802	monocyte_LPS
ENSG00000188011	RTP5	2.879666313	1.03183	CD8_T-cell_anti-CD3-CD28
ENSG00000188011	RTP5	8.25559435	0.908154	monocyte_IAV
ENSG00000188011	RTP5	8.655446615	0.632605	monocyte_naive
ENSG00000261186	LINC01238	1.646953613	-0.511456	Th1-17_memory

Reference

1. Ascha, M.S.; Hanouneh, I.A.; Lopez, R.; Tamimi, T.A.; Feldstein, A.F.; Zein, N.N. The incidence and risk factors of hepatocellular carcinoma in patients with nonalcoholic steatohepatitis. *Hepatology* **2010**, *51*, 1972-1978.
